# Tetraspanins: Physiology, Colorectal Cancer Development, and Nanomediated Applications

**DOI:** 10.3390/cancers13225662

**Published:** 2021-11-12

**Authors:** Stefan Titu, Cristiana Maria Grapa, Teodora Mocan, Ovidiu Balacescu, Alexandru Irimie

**Affiliations:** 1 “Iuliu Hatieganu” University of Medicine and Pharmacy, Faculty of Medicine, 400126 Cluj-Napoca, Romania; stefan.titu@umfcluj.ro (S.T.); grapa.cristiana.maria@umfcluj.ro (C.M.G.); airimie@umfcluj.ro (A.I.); 2 Department of Surgical Oncology, The Oncology Institute “Prof. Dr. Ion Chiricuta” Cluj-Napoca, 400015 Cluj-Napoca, Romania; 3 Nanomedicine Department, Regional Institute of Gastroenterology and Hepatology, 400126 Cluj-Napoca, Romania; 4 Department of Genetics, Genomics and Experimental Pathology, The Oncology Institute “Prof. Dr. Ion Chiricuta” Cluj-Napoca, 400015 Cluj-Napoca, Romania; ovidiubalacescu@iocn.ro

**Keywords:** tetraspanins, physiology, colorectal cancer, nanotechnology

## Abstract

**Simple Summary:**

Considering the high incidence of colorectal cancer in adults, as well as the need for identifying novel therapies, we hereby explore the role of tetraspanins in the development of colorectal cancer. We have focused on variate aspects starting from the structure and general physiology and ending with the precise mechanisms involved in the dual reported role of tetraspanins (pro–tumoral and tumor suppressor key player element). Moreover, the present review focuses on the potential of tetraspanins as a target for nanotechnology-mediated therapies, also gathering the limited attempts towards this aim and their reported data.

**Abstract:**

Tetraspanins are transmembrane proteins expressed in a multitude of cells throughout the organism. They contribute to many processes that surround cell–cell interactions and are associated with the progress of some diseases, including cancer. Their crucial role in cell physiology is often understated. Furthermore, recent studies have shown their great potential in being used as targeting molecules. Data have suggested the potential of tetraspanins as a targeting vector for nanomediated distribution and delivery for colorectal cancer applications. Our aim is to provide a review on the important part that tetraspanins play in the human organism and highlight their potential use for drug delivery systems using nanotechnology.

## 1. Introduction

**Figure 1:**
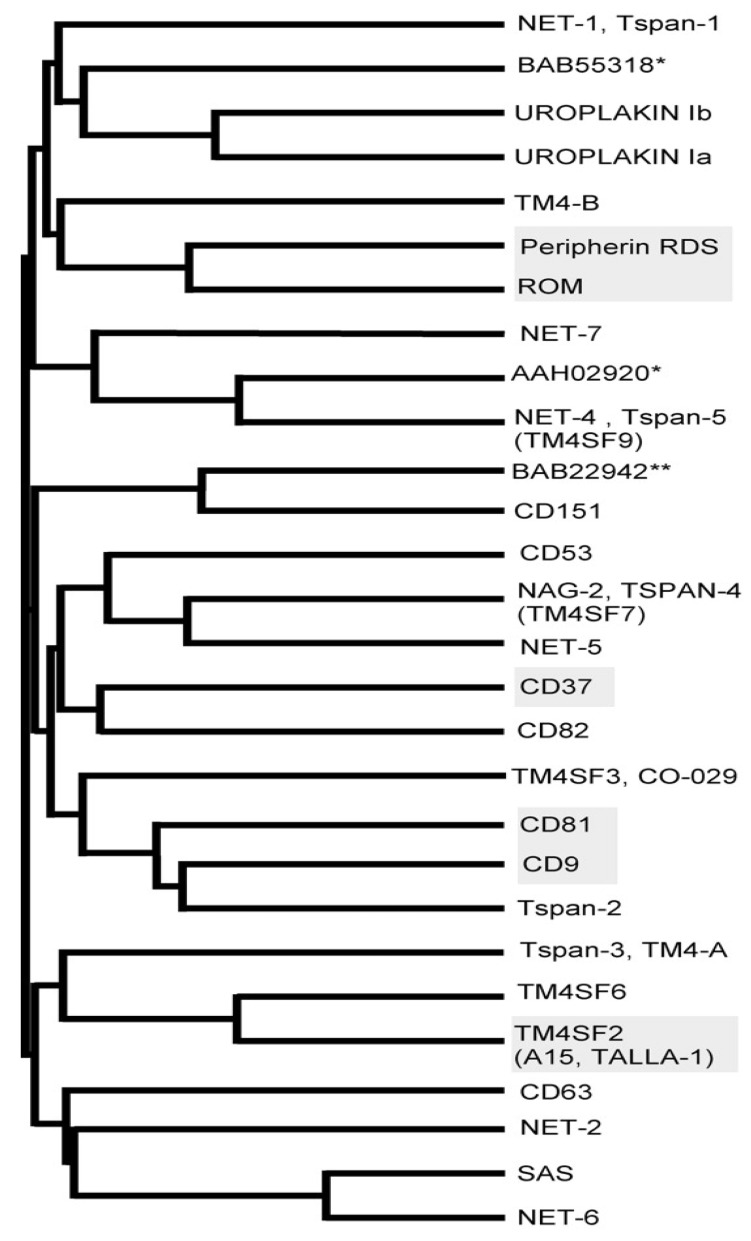
Mammalian tetraspanin family. The 28 family tetraspanin members. The shaded tetraspanins represent the ones that are either mutated in humans or deleted in mice. Reprinted with permission from [7]. * Accession number—no other names available. ** Murine sequence.

Transmembrane proteins contribute to numerous cell functions, from their development to pathological processes like tumor cell proliferation or metastasis. Tetraspanins are included in the transmembrane protein’s family, which includes 33 members in humans, that are highly expressed in different types of tissues and cell types, of which 28 members are represented in Figure 1. In addition, 37 more members were identified in Drosophila melanogaster sp. as well as 20 more in Caenorhabditis elegans sp. [1]; they are implicated in cell adhesion, migration, tumor proliferation, or immune activation [2,3].

Through interacting with each other or other proteins, tetraspanins have the ability to organize protein complexes named tetraspanin-enriched membrane domains (TEM), which play a role in the compartmentalization of different types of receptors, lipids, or signaling molecules. This organization is called the “tetraspanin web”. Some examples of the most important non-tetraspanin affiliates are integrins, such as CD29, CD49, or CD104; matrix metalloproteinases; immunoglobulins (ICAM-1, VCAM-1, EpCAM); ADAM10; or E-cadherin; and many more molecules [4].

Regarding tetraspanins’ function, while conclusive studies are lacking, with the help of antibodies used against this type of proteins, a rather clearer image has been painted: studies on their distribution, association, and function have pointed out their role in cell activation, proliferation, differentiation, cell adhesion or motility, and cancerous transformation [5]. Additionally, tetraspanins have been proven to interact with each other through various mechanisms, including palmitoylation [6].

The process of palmitoylation contributes to the web formation of tetraspanins. It also influences their signaling and cell morphology. Three studies regarding tetraspanin palmitoylation showed that in CD9 and CD151, the intracellular cysteine residues that are proximal to the four transmembrane domains are all palmitoylated. The loss of this process leads to fewer tetraspanin–tetraspanin interactions. Still, the process of palmitoylation alone is not sufficient for tetraspanins to establish interactions with other proteins [8].

Other studies involve tetraspanins in different cellular processes, such as cell–matrix interactions, cell–cell fusion, cell migration, differentiation, or even immune cell activation. The multitude of roles attributed to tetraspanins is due to their presence on almost all cell types [6].

Nanotechnology is a vast domain that entails the use of nano-sized materials in order to improve diagnosis or treatment for numerous diseases. Nanoparticles, such as gold, silver, dendrimers, carbon nanotubes, and others, have been successfully used for imaging, targeted delivery, and treatment in various types of pathologies, with promising results. In order to achieve a proper accumulation of the nanoparticles in the desired cell or tissue, targeting molecules, such as antibodies or ligands, are used, including tetraspanins, which have shown encouraging potential.

The main purpose of this narrative review is therefore to characterize tetraspanins and their role, with a special focus on their implication in cancer. Additionally, we aim to review the targeting role of tetraspanins in connection to various nanotechnological applications, by gathering already published data on nanomediated applications and focusing on their potential use in the field of nanomedicine.

## 2. Structure and Function

**Figure 2:**
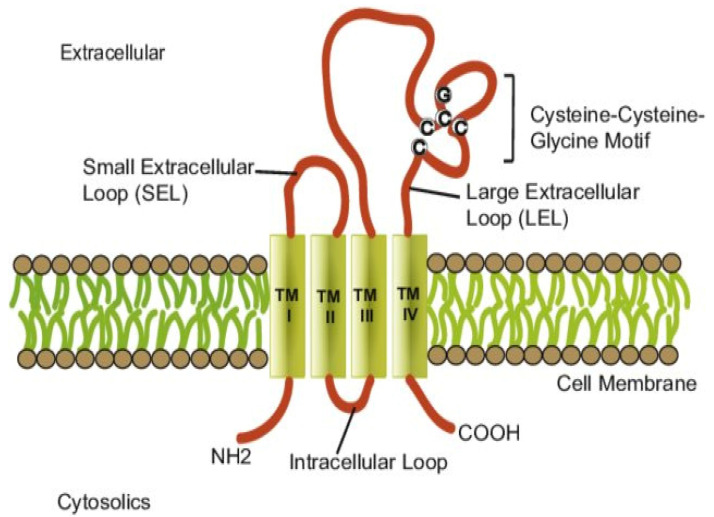
Tetraspanin structure: Four transmembrane domains (TMI-IV), a small extracellular loop, a large extracellular loop, and the intracellular loop with C-terminal and N-terminal regions. Reprinted with permission from [10].

For tetraspanins, four transmembrane domains (TMs) have been described: the first two domains flank a small extracellular loop (SEL) while the last two domains flank a large extracellular loop (LEL) (Figure 2). They also contain conserved cysteine-cysteine-glycine residues, which have the ability to develop disulfide connections with other cysteines. The four TM domains also delimit three cytosolic domains [6,9]. The cytosolic N- and C-terminal extends include well-preserved polar residues; these residues are of utmost importance in creating stability and also seem to play a part in the transmembrane helices’ interaction [2].

The C terminal region appears to play an important part in the interaction between proteins, seeing as it differs from one tetraspanin to another, while for the N terminal region, no considerable function has been described. PDZ domains that bind to the C-terminal region of several tetraspanins have also been described, including the interaction between CD63 and syntenin’s PDZ domain and EPP50 or Sap97 proteins, which are highly expressed in the retina of rats and interact with CD81 [2].

Studies regarding the small and large extracellular loops have so far pointed out that the LEL contains crucial sites of recognition and cell interaction, while SEL appears to play a less important part. One study involving the binding of hepatitis C virus to CD81 demonstrated that LEL, but not SEL, was required for the binding process. The small domain was only required for the optimal expression of the LEL. Another study pointed out that some monoclonal antibodies exclusively recognize only LEL and not SEL [3].

It is believed that an important part in tetraspanin–tetraspanin interaction is mediated through the hydrophobic connection between the TM domains: TM1, TM3, and TM4 contain high polar residues (asparagine, glutamine, glutamic acid), which are conserved in 70–90% of all tetraspanins. These polar residues have the ability to develop solid hydrogen bonds, therefore leading to a stabilized TM tertiary structure. For a proper biosynthesis of the tetraspanins, the interaction and orientation of the TM domains is crucial. Therefore, their packing process can represent a checkpoint for tetraspanin development, including the process of extracellular domain folding [8].

Some tetraspanins, like CD81, play an important part in the immune system. Studies on its large extracellular domain have delivered indispensable insight regarding its structure and function. It appears that the LED of CD81 comprises a variable domain inserted into a conserved domain, all stabilized by disulfide bonds with different numbers of cysteine residues. Furthermore, it has been discovered that its small extracellular domain (SED) is compacted into a loop in the LED, and the transmembrane region forms a coil-shaped structure stabilized by hydrogen ties. Tetraspanins also go through a glycosylation process to fluctuating degrees and are post-translationally changed by adding palmitate fractions to their cysteine residues [1].

Tetraspanins have also been linked to enhanced cancer progression and metastatic behavior. It appears that high tetraspanin levels, namely CD82 and CD9, are related to tumor growth inhibition, as studies have demonstrated that in some late-stage tumors, such as colorectal cancer, these tetraspanins are downregulated and therefore associated with poor prognosis [4]. Additionally, high levels of CD9 and CD82 have been associated with a favorable prognosis in different types of cancers, like breast, pancreas, prostate, and lung cancer [6].

TSPAN8, as a member of the tetraspanin family, has been demonstrated to be overexpressed in lung, pancreatic, gastric, colorectal, and liver cancers. Its contribution to cancer cell proliferation, survival, metastasis, and tumor angiogenesis has been well documented. Unfortunately, no study so far has been able to validate it as a therapeutic target [11]. Additionally, while it appears that the role of CD9 in promoting or inhibiting tumor progression is rather controversial, CD151 and TSPAN8 are believed to be involved in tumor progression, displaying rather oncogenic properties. CD82, on the other hand, plays a part in metastasis suppression [12].

Excluding erythrocytes, most cells have the ability to express tetraspanins [13]. Regarding their tissue distribution, given the fact that only a small number of tetraspanins have been properly studied, no specific data can be extracted but rather a general knowledge of the subject. It appears that while some tetraspanins are widely distributed throughout the organism, some of them are restricted to specific areas. One particular example is given by uroplakins UP1a and UP1b or RSD/peripherin and ROM-1, proteins found only in the urothelium membranes and the photoreceptor segment disc, respectively [14,15].

Other types of tetraspanins are also limited to specific areas regarding their expression levels: CD53 is only expressed by leukocytes, while CD37 is used as a marker for lymphoid B cells [16]. Additionally, while some tetraspanins are only expressed on the surface of certain cells, others can be observed in the intracellular compartments: CD63, as an example, has been identified in the storage granules (Weibel–Palade bodies) of the endothelial cells, in the lysosomes of platelets, and in the azurophil granules of neutrophils [17,18].

Furthermore, tetraspanin’s behavior has been studied for the past years. Analyzing one single molecule of CD9 using total internal reflection fluorescence (TIRF) microscopy showed that it cycles between tetraspanin-enriched microdomains (TEMs) [3] and the rest of the membrane. CD9 has a confined motion at the basal cell surface and reveals a lower diffusion rate and this is why these enriched areas correspond to domains of the membrane where several molecules are confined synchronously. Besides these regions, CD9s is in a Brownian motion [1].

Looking forward to tetraspanins like CD53 and CD63, it was found that they are expressed in the major histocompatibility II class-enriched compartments (MIIC) of B lymphocytes, especially in the internal membranes and in the MIIC multivesicular exosomes. It was discovered that CD63 and CD82 in MIIC are connected to various human leukocyte antigen (HLA) molecules [19].

## 3. General Physiology

In the process of interacting with other molecules, it seems that tetraspanins first pair up with partner proteins and then connect them to other tetraspanins or their partners, as demonstrated by a study that offered a clear description of the interaction between CD151 and integrins or CD9 and its partner, CD9P-1. Another important role is represented by their contribution to the stoichiometry of all the molecular connections that occur in the tetraspanin web or the control of their protein partners [1].

When talking about tetraspanin interactions, it is important to mention that tetraspanin–tetraspanin exchanges are weaker than interactions with other different partners, for example, between them and other integrins. This type of contact with different types of integrins may contribute to the important role tetraspanins play in the process of tumoral migration and metastasis. As an example, the protein kinase C (PKC) family of enzymes, which control other protein’s function through the phosphorylation of serine and threonine residues, has the ability to interact by binding indirectly to other tetraspanins (like CD9,82 or CD 151) and therefore contribute to other biological events. For most of these connections, tetraspanins act as linker molecules, by engaging PKC proteins with integrins [20].

Examples of tetraspanins with a main function of regulating the trafficking of their partner proteins have emerged. As an example, the main purpose of CD63 is believed to be related to enabling the internalization of other partner proteins or facilitation of their aim of endocytic organelles [1]. Examples of these types of proteins include the synaptotagmin 7, which plays a part in regulating lysosome exocytosis and therefore in the repairment of the plasma membrane [21].

Another example is provided by the Drosophila Sunglasses tetraspanin. It is primarily expressed in the retina, and it encodes a lysosomal protein, which is involved in the endocytosis and degradation of the light receptor rhodopsin. Its lack of expression leads to retinal degeneration, which is light dependent [22].

Tetraspanins also have the ability to modulate their partner protein’s trafficking on the secretory pathway. As an example, a study uncovered that a group of tetraspanins called TSPANC8 have the ability to regulate the metalloproteinase ADAM10’s exit from the endoplasmic reticulum and stimulate its accumulation at the cell surface. The tetraspanins therefore induce Notch activation and signaling, by activating Notch receptor through ADAM10 release [23].

Another role for CD81 was revealed by a study on a case of immunodeficiency in a young patient. A mutation of CD81 that can lead to an important decrease in levels of CD19 seemed to be involved in this case. CD19 is an important partner for CD81 and is essential in stimulating B lymphoid cells, so a reduction in its activation due to CD81 mutation can lead to this type of disorder [24].

Besides neoplastic tissue, endothelial cells also express numerous types of tetraspanins, like CD9, CD81, oandr CD151, and subgroups like TSPAN4 and TSPAN8. These tetraspanins play an important role in angiogenesis, vascular development, and leukocyte identification [20].

Another role for a tetraspanin–TSPAN12 was described through its interaction with Wnt receptor Frizzled-4 or the ligand Norrin. It is a known fact that mutation of this receptor leads to a disease called familial exudative vitreoretinopathy (FEVR) in humans. Additionally, seeing as TSPAN12 is overly expressed in the retina, the effect of the tetraspanin on Wnt signaling became a subject of interest. Researchers showed that TSPAN12 is almost exclusively expressed in the retinal vasculature, and it interacts with the ligand Norrin, promoting Norrin b-catenin signaling but not Wnt/b-catenin induced signaling. The main purpose of the study was to prove that mutation in this specific type of tetraspanin could cause/contribute to the development of FEVR [24].

Additionally, tetraspanins like CD9, CD 82, and CD151 are related to the signaling process of tyrosine kinase receptors, such as the epithelial growth factor receptor (EGFR) family, c-MET, and endothelial growth factor receptor (VEGF) [25]. However, there are still studies lacking in regard to which particular tetraspanin is directly associated with these types of receptors. Some studies indicate that CD151’s involvement in the signaling process is due to its coupling with certain integrins and that for CD82, its involvement is indirect [26].

Other tetraspanins have been implicated in the regulation of protein tyrosine kinase 2 and focal adhesion kinase (FAK), most often involved with the process of cellular adhesion. Studies proved that siRNA knockdown of CD151 led to reduced phosphorylation of FAK. Additionally, treatment with a monoclonal antibody against CD151 led to the same effect. In order to prevent this effect, researchers tried to treat the cells with a β1 integrin-activating antibody, but the phosphorylation process was not improved even with forced integrin activation. This study came to the conclusion that tetraspanins may be involved in the integrin-mediated signaling process regardless of the initial activation of the integrins [27].

A study conducted by Naoyouki et al. revealed that some tetraspanins, like CD9, CD63, and CD81, are associated with the stem cell factor receptor (c-Kit) or kit ligand. After ligand binding, c-Kit signaling is activated, which, in turn, activates other signaling proteins, like PI3K, MAPK, and Src. The interaction between tetraspanins and c-Kit was also enhanced after treatment with its ligand, namely, stem cell factor [28]. Additionally, there is a possibility that CD63 could stabilize c-Kit or even modulate the signaling cascade by altering the process of protein trafficking. This process could significantly impact certain types of leukemias, in which c-Kit expression or the activation process is dysregulated [29].

Cell morphology is highly regulated by cell–cell interactions, and integrins are known to be a part of this process. It appears that integrins like β-catenin are not only receptors for the extracellular matrix but are also involved in cell–cell adhesion. Chattopadhyay et al. demonstrated that a complex formed by associating tetraspanin CD151 with α3β1 integrin is involved in cell–cell interactions by promoting cadherin-mediated adhesion by modifying the levels of gene expression. Furthermore, levels of CD65 were also related to β-catenin signaling. It appears that high tetraspanin levels can stabilize the signaling process. These findings are important because they show the crucial role that tetraspanins play in cell morphology and behavior [30].

## 4. Tetraspanins in Cancer: Malignancy Role

As stated, tetraspanin family members are involved in the development of neoplastic tissue by regulating cell adhesion, proliferation, metastasis, and angiogenesis [19,20]. The motility of a tumoral cell is important for the process of cancer invasion or the appearance of metastasis (Table 1). The loss of interactions like cell–cell adhesion and the upregulation of cell–matrix interactions leads is complex and multifactorial, requiring the participation of different types of molecules. The main contributors are represented by adhesion proteins, like integrins and E-cadherins; growth factor receptors, like EGFR or VEGF; proteinases; cytoskeletal proteins; and, of course, cell membrane proteins like tetraspanins [31].

An early step in cell migration is the process of actin reorganization. It appears that tetraspanins contribute to this process by regulating the dynamics in the development of cytoskeletal actin [20].

Integrins, through their role in cell morphology, also contribute to cancer progression by interacting with tetraspanins. For example, a study showed that by upregulating α5β1 integrin expression, apoptosis can be inhibited and β1 integrins can stimulate metastasis [32]. The process by which β1 integrin promotes cell invasion is represented by the formation of adhesion complexes with the ECM. It also has the ability to activate the signaling cascade Akt/phosphoinositide 3-kinase (PI3-K), which also contributes to the activation of the adhesion kinase [33].

Another study on a breast cancer cell line, namely MDA-MB-231, demonstrated that the complex formed by α3β1 integrin with tetraspanins is associated with an invasive tumor phenotype. The reason for this association is due to the activation of numerous signaling cascades, like PI3-K and MMP-2, a metalloproteinase, which is related to the promotion of migratory patterns for tumoral cells [34]. One more integrin, namely αvβ4, through activation, can contribute to breast cancer cell proliferation and metastasis, as shown in a study on breast cancer cell lines and a mouse model [35].

Despite the large diversity among tetraspanins, some have been clearly demonstrated to play key roles in cancer, among which CD151 and CD9 represent the most important ones [36]. Interestingly, although most tetraspanins are downregulated during the process of metastasis, the CD151 tetraspanin is the first to be associated with the promotion of this process [37]. Similarly, the TSPAN8 family was found to be upregulated in many types of cancers, like colorectal, gastric, esophageal, hepatic, and pancreatic carcinomas [38]. CD9, another tetraspanin, which was believed to be downregulated in most types of cancers, was demonstrated to promote cancer progression for prostate cancer, breast cancer, and osteosarcoma [20]. These findings are also endorsed by the fact that CD9 can promote the expression of MMP-2, a proteolytic enzyme [39]. The dual role of tetraspanins as anti-oncogenic and pro-metastatic is currently under intense research, as recent papers suggest [40].

**Table 1:** Role of tetraspanins in malignancies.

Tetraspanin	Type of Cancer	Mechanism of Action	References
TSPAN 1	Colon cancer Squamous cells carcinoma Hepatocellular carcinoma	Promoter of invasion but the mechanism has not been determined.	[41,42]
TSPAN 8	Colorectal cancer	TSPAN8 suppresses the motility of cells by regulating the tumor cell–matrix and cell to cell adhesion in colorectal cancer. At the same time, regulation by the E-cadherin/p120ctn complex has been found to be a promoter of invasion in colon cancer.	[43,44]
TSPAN 13	Breast cancer	Cell motility in breast cancer is suppressed by downregulation of the matrix metalloproteinases.	[45]
CD9	Ovarian cancer Fibrosarcoma Breast cancer	Suppressor of cell motility in ovarian carcinoma by modifying cell adhesion on the extracellular matrix; altered integrins (β1, α2, α3β1, α5, and α6) were found due to downregulation of CD9. Suppressor in fibrosarcoma by cell motility inhibition through CD9 complexes formed with TGFα, EGFR, EWI-2, EWIF, and β1. Promoter of cell motility in breast cancer by promoting α3β1 integrin.	[46,47,48]
CD63	Colon cancer	Suppressor in colon cancer by regulating cell adhesion and migration.	[49]
CD81	Breast cancer Histiocytic lymphoma	Promoter of cell motility by promoting α3β1 integrin in breast cancer. Promoter in histiocytic lymphoma through cell membrane structures.	[48,50]
CD82	Ovarian cancer Non-small lung cancer	Suppressor in ovarian cancer by inhibiting αvβ3 integrin/vitronectin-mediated cell motility and proliferation. Suppressor in lung cancer by regulation of β1 integrin maturation.	[51,52]
CD151	Gastric cancer Breast cancer Hepatocellular carcinoma	Promoter in gastric cancer in association with α3 integrins. Promoter in breast cancer in association with integrins promoting a signaling pathway (HGF/c-MET). Promoter in liver cancer by increasing Rac/Cdc42 activity.	[53,54,55]

### 4.1. Tumor Progression-Promoting Tetraspanins

#### 4.1.1. TSPAN 8

The TSPAN8 tetraspanin was initially recognized as a tumor-associated antigen, but further studies proved that its expression is highly correlated with the progression of cancer cells [38]. As stated above, numerous types of cancers, mainly of the gastrointestinal tract, have been associated with a high level of TSPAN8 expression (Table 2). The levels are also associated with advanced stages and poor prognosis in these types of tumors [56]. One study showed that this type of tetraspanin has the ability to promote intrahepatic metastasis of hepatocellular cancer cells. Furthermore, it was shown that cancer cells that express TSPAN8 metastasize uncontrollably and do not stop at the lymph nodes. A study on a pancreatic cancer cell line showed that TSPAN8 expression leads to the development of disseminated intravascular coagulation and hemorrhage at the tumor site, as well as in metastatic tumors [57].

In pancreatic and colorectal cancer cells, it appears that CD151 associates with TSPAN8 and integrin a6b4, the complex leading to PKC activation [58].

#### 4.1.2. CD151

The role of tetraspanin CD151 in the development of cancer metastasis has been well documented so far (Table 2). Furthermore, this tetraspanin was also linked to various other diseases, like nephrotic syndrome, primary glomerular disease, and even the process of wound healing [20]. In cancer, a high level of CD151 is correlated with a grim prognosis for patients, and with elevated recurrence rates [59].

A study by Zijlstra et al. showed that one of the mechanisms involved in CD151’s ability to enhance tumor metastasis is through the direct boost of cellular movement. Increased expression of CD151 in vitro leads to high tumor cell invasion and proliferation, while low levels are associated with inhibition of these cellular processes [60].

Other recent studies regarding CD151 expression revealed that its level is correlated with tumor neovascularization, with the tetraspanin’s levels being directly connected to the angiogenesis process of neoplastic tissue [59]. Additionally, the process of proteolysis and enabled signaling, which is CD151 dependent, is likely to promote tumor progression [38]. Therefore, it is implied that CD151 acts on multiple layers and its inhibition could potentially be useful for the development of oncologic treatments.

### 4.2. Tumor Progression-Suppressing Tetraspanins

#### 4.2.1. CD9

Regarding tetraspanin CD9, several studies showed that its knockdown is correlated with the promotion of tumoral invasion and progression, in several types of cancers, such as head and neck, skin, ovary, prostate, colon and stomach, and others [61]. A low expression of CD9 was noted for metastatic tumors, when compared with primary lesions (Table 3). A lack of CD9 in patients was also seen in advanced stages of the disease. However, although some authors proposed CD9 knockdown as a metastatic suppression mechanism in MDA cells in mouse xenografts [61], other groups reported opposite results, namely enhanced integrin-dependent adhesion and inhibition of cell growth in the case of ectopic expression of CD9 in colon carcinoma cells [62]. This association of CD9 with various coupling proteins is especially considered as the mechanism for variate or even opposite effects, with pro-metastatic functions also being reported [63].

However, while there is much more information needed in order to establish the prognostic significance of CD9 in cancer, most studies agree on the fact that its expression is related to patients’ survival rates, disease-free interval, or recurrence rates [64,65]. Additionally, due to its presence in the lymphatic vessels, CD9 may also be involved in tumor–endothelial cell interaction and therefore contribute to tumor lymphangiogenesis [66].

Studies on tetraspanin’s effect on cancer cells’ motility have established that the CD9-integrin complex contributes to cell–extracellular matrix adhesion mediated by integrins; however, CD9 also regulates cell–cell interactions and promotes cytoskeletal organization, leading to cellular restructuring, so its impact on cell morphology and its understanding have not yet been clearly established [67].

As mentioned, it is clear that CD9 is involved in tumoral proliferation and metastasis by suppressing these processes, but more research is needed in order to fully understand its role and possibly its therapeutic potential.

#### 4.2.2. CD63

CD63 cDNA was transfected into human melanoma cell lines in a study by Hwa-In et al. They wanted to reveal whether CD63 would stimulate or inhibit the tumor cells. In vitro studies performed showed that downregulating CD63 stimulated progression and metastasis, which is in accordance with previous studies [62].

Other types of cancer that progress when CD63 levels are low are represented by lung and ovarian cancer (Table 3). Other types like prostate, pancreas, and thyroid cancer are not related to CD63’s downregulation [68].

#### 4.2.3. CD82

The levels of the tetraspanin CD82 are indirectly proportional to the patient’s prognosis in many types of cancers, like gastric, colorectal, lung, breast, bladder, prostate, and endometrial cancer and others [69]. It was discovered that in estrogen receptor-positive breast cancer, CD82 levels were very low, but in estrogen receptor-negative tumors, the levels were normal [70].

It appears that the mechanism by which CD82 contributes to cancer progression is by altering the function of different types of molecules, such as integrins, growth factor receptors, c-MET, and uPAR (Table 3). CD82 also has the ability to interact with different molecules in the tumor microenvironment [71]. CD82 also has the ability to interact with an EGFR, namely alpha6 integrin, which leads to an altered laminin adhesion or migration process. The tetraspanin can also play a part as a tumor suppressor by influencing the activity of EGFR. High levels of CD82 can lead to increased internalization of the growth factor receptor, thus inhibiting its signaling cascade. CD82 was also found to suppress the ubiquitination of EGFR, leading to altered endocytic trafficking of transmembrane cargos [72].

### 4.3. Tetraspanins in Colorectal Cancer

Colorectal cancer (CRC) is one of the leading causes of death in the world, and one of the most common types of cancer pertaining to the gastrointestinal tract. There are over one million new cases every year, and unfortunately, studies have shown that this is an increasing trend, and the age of occurrence also tends to be lower due to changes in diet and lifestyle [73]. As stated before, TSPAN8 is one of the tetraspanins that is positively correlated with colorectal cancer, with its high expression being linked to low chances of survival in this particular category of patients. Studies have shown that its expression is increased in CRC, that it can be used as a marker for targeted therapy, and it correlates with drug resistance in CRC treatment. A study also showed that TSPAN8 levels promote the progression of cancer stem cells in CRC, a process that is dependent on β-catenin expression [74].

Other studies implied that TSPAN8’s large extracellular loop could be involved in the progression of CRC and could also represent a prospective therapeutic target. A group of researchers [11] used several competition assays with the LEL of TSPAN8 on a CRC cell line along with its knockdown to prove that the LEL is essential in augmenting cancer cell invasion. By creating an antibody directed to the large extracellular loop of TSPAN8, the authors also demonstrated that antibody-based inhibition of TSPAN8-LEL could significantly inhibit the invasiveness of CRC cell lines. The team thus proposed that TSPAN8-LEL could represent a potential therapeutic target for CRC treatment [11].

It is a known fact that exosomes are vesicles released into the extracellular space by numerous cells. Some tetraspanins are found on the surface membrane of exosomes. Among them, CD9, CD63, and CD81 are most frequently found on the surface of exosomes. Therefore, they could be used as biomarkers for the detection of certain exosomes. Based on this fact, a team of researchers used three colorectal cancer cell lines and examined the exosomes derived from them through Western blotting. The CRC cell lines used were HCT-15, SW480, and WiDr. From the tetraspanins enumerated, CD81 was the only one that was found in all three cancer cell lines. CD9 was found in the exosomes coming from the HCT-15 and SW480 lines and CD63 from the HCT-15 line. The study concluded that CD81 could represent a potential biomarker for exosomes that originate from CRC cell lines [75].

Studies regarding the role of CD151 in CRC are inconclusive. While some suggest that this tetraspanin is associated with the metastatic phenotype in CRC, others found that CD151’s expression is lower in CRC tissue compared with normal surrounding tissue. These studies could indicate a dynamic role for tetraspanin’s expression in CRC. Peng-Chan et al. used tissue samples from 118 patients with CRC and determined the presence of CD151 through tissue staining. They discovered that patients with metastatic disease or patients with tumors that were highly invasive presented with low expression of CD151. For patients in the early stage, the level of CD151 was not associated with overall survival. The explanation for this fact could be related to the hypoxia present in late stages, which can lead to a decrease in tetraspanin expression. The study concluded that low CD151 expression is correlated with metastatic disease [76].

Levels of CD63 were also associated with a poor prognosis in CRC for a subgroup of patients who presented with metastatic disease. CD63 was found to be an independent predictor for a negative prognosis in colorectal cancer in general and also for patients with metastatic disease. A study on 620 consecutive CRC patients found using immunohistochemistry analysis that CD63 levels were correlated with adverse outcomes in a directly proportional manner. The study also suggested that high expression of tetraspanin is related to the epithelial-to-mesenchymal transition (EMT) phenotype that occurs in later stages and is related to worse outcomes for cancer patients [77].

TSPAN6 was also detected in CRC tumors with the help of tissue staining. It was discovered that compared to the center of the neoplastic tissue, the staining was lower compared to the other areas of the tumor, where the density of the tetraspanin was higher. Additionally, studies found that TSPAN6 was significantly less expressed in cancer cells, compared to normal tissue, suggesting that the loss of expression promotes CRC progression [78].

## 5. Nanoparticles and Extracellular Vesicles

In regard to different methods of cell–cell communication, extracellular vesicles (EVs) have surfaced as a new method. They are seemingly involved in a multitude of biological or pathological processes. They can be extracted from all types of body fluids like urine, saliva, blood plasma, cerebrospinal fluid, amniotic fluid, ascites, etc., and recent studies have proposed a new role for EVs in regard to gene transfer. EVs are composed of activated proteins, lipids, and mRNA; therefore, by transferring their composition into a target cell, it can lead to altered gene expression, eventually influencing the cell’s activation of the differentiation process [79,80]. It was demonstrated that EVs can influence the metastatic development of certain tumors, activate endothelial cells, induce the progression of certain neurodegenerative diseases, and therefore, participate in all kinds of physiological and pathological processes [79,81,82]. They also have the ability to induce regeneration of certain tissue samples or mediate immune reactions. All these characteristics made them ideal for studies regarding their therapeutic applications. A particular subtype of EVs are represented by exosomes, which have a unique protein profile [83,84].

The contribution of EVs to the evolution of tumoral cells to a metastatic phase is represented by the crosstalk between the vesicles pertaining to the cancer cells and the surrounding cells in the neoplastic tissue. One of the main steps in this interaction is the migration and invasion of the cells, which takes place in the extracellular matrix [83].

Tetraspanins like CD9, CD63, and CD81 are often used as biomarkers for extracellular vesicles, but it is still unknown what effect their change of expression has on EVs’ function or composition. They are essential to the process of formation and recruitment of EVs and studies have shown that altered tetraspanin expression can influence the functionality of EVs [83].

Many more studies are still required in order to properly define the role of tetraspanins in determining EV biology [72].

**Figure 3:**
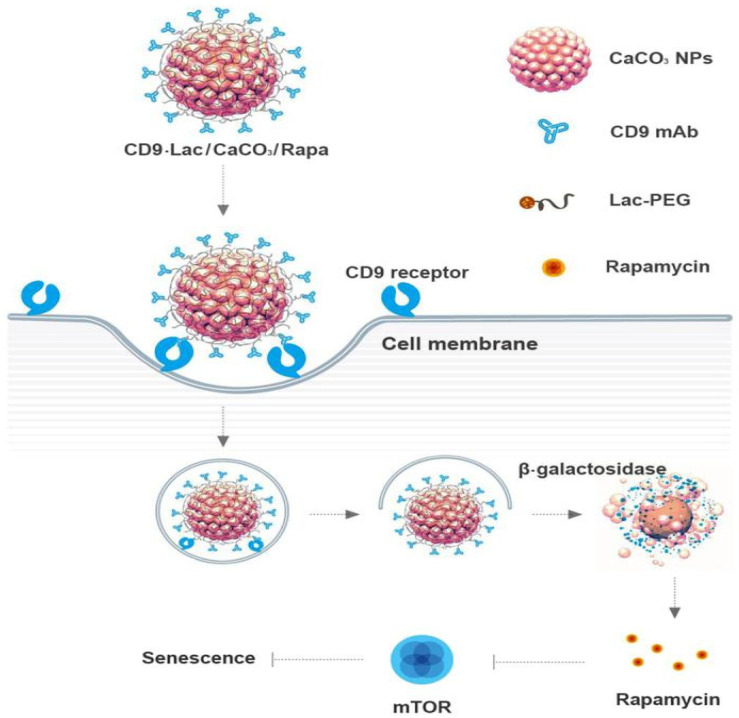
Calcium carbonate (CaCo3) nanoparticles with the tetraspanin CD9, Rapamycin, and lactose. Rapamycin was enabled due to the β galactosidase activity of cleating lactose. Reprinted with permission from [85].

Regarding the use of tetraspanins in nanomedicine, studies so far have given promising results. A CD9 monoclonal antibody was used together with Rapamycin, an mTOR inhibitor, which was found to inhibit the process of senescence, and calcium carbonate nanoparticles, in order to target senescent cells. Rapamycin was released in vitro through β galactosidase activity, by unwrapping the lactose surrounding it. The CD9 tetraspanin was found to be overexpressed by senescent cells, so its use was highly justified. The nanocomplex showed good results, with high uptake and promising anti-senescence results, suggesting that it could represent a potential tool for the treatment of some aging-related diseases (Figure 3) [85]. The same team also demonstrated that PEGylated liposomes together with CD9 antibodies and Rapamycin can be successfully used for senescent cell targeting, although further in vivo studies are required [86]. On the same note, another team of researchers used mesoporous silica-coated nanoparticles, coated with hyaluronic acid and functionalized with CD9 antibodies and rosuvastatin as a drug, in order to target senescent atherosclerotic plaques, which are mostly made up of endothelial cells and macrophages. The use of CD9 antibodies led to a high uptake of the nanocomplex and overall, promising therapeutic use [87].

An interesting approach for infarcted heart tissue treatment was used by Liu and Co-authors in 2020 [88], who based their experiment on the fact that the distribution of EVs is central for tissue homeostasis. They used magnetic nanoparticles conjugated with CD63, which is found on the surface of EVs, in order to target the injured cardiomyocytes. With the use of a magnetic field, the nanoparticles released the EVs, which expressed CD63, and this led to a reduction in the infarcted area and improved left ventricular ejection fraction. The authors also noted that this approach could also have potential use in other diseases.

Polymeric nanoparticles were loaded with CD63-targeting antibodies in a study on monocyte-derived dendritic cells. The team also used a pre-adsorption process for attaching the targeting antibody on the surface of the nanoparticle. The process was demonstrated to play an important role in improving surface targeting, seeing as once a nanocomplex is introduced in a biological fluid, through the process of protein adsorption, they develop a protein corona, which impedes proper targeting by covering the whole nanocarrier. The study confirmed that the pre-adsorbed antibodies could better target the cells of interest, proposing a new method in the field of precision medicine [89].

A nanoparticle-based immunoassay was developed by Khirul et al., with their purpose being better detection of extracellular vesicles. The team used antibodies against tetraspanins conjugated on nanoparticles in order to detect EVs from cell cultures or urine samples. Urinary EVs could play an important part in the detection of certain urinary-tract tumors, so their quick and specific detection using nano-based approaches could be very useful, if applied correctly [90].

A trapping protein was designed using the LEL of CD81 and streptavidin, and then loaded onto polysaccharide-based nanoparticles. The system’s role was to trap the hepatitis C virus, seeing as the virus uses the LEL of CD81 to recognize the hepatocytes and enter the cells through receptor-mediated endocytosis. The nanocomplex developed showed promising results, being able to reduce viremia both in vitro and in vivo, and thus offering a new possible treatment for hepatitis C [91].

Nanotechnology has also proved useful for engineering hybrid small-sized EVs, also called exosomes. Seeing as EVs are usually difficult to isolate through standard isolation techniques, and subsequently be used for different cell-targeting procedures, a team of researchers used synthetic liposomes, which were further hybridized with EVs from mouse macrophages. In order to identify the exosomes, standard blot techniques were applied to recognize CD9, CD81, and CD63 as surface biomarkers. The last step was adding doxorubicin and administering the nanocomplex to cancer cells. The complex developed showed enhanced tumor cell toxicity. The authors therefore proposed this nanosystem as a potential drug cargo for targeted delivery in various types of cancers [92].

EVs themselves have proved to be good drug carriers, so combining them with nanoparticles would only increase the efficacy for a drug delivery system. In 2021, Niu and co-authors [93] developed a nanocomplex based on heparin-based nanoparticles, loaded with doxorubicin, and then patched with extracellular vesicles, in order to improve doxorubicin’s efficacy on glioma cells. The complex managed to surpass the blood–brain barrier and accumulated in a high amount at the tumor site, thus offering another potential use for extracellular vesicles.

## 6. Conclusions

Tetraspanins are implicated in a multitude of cell signaling pathways, play a crucial role in cell–cell interactions, and have dysregulated expression levels in many types of cancers. Further studies are necessary in order to gain a better understanding of their role and potential use. Regarding drug delivery systems, most drugs that are clinically approved still have wide limitations due to their lack of specificity when accumulating at their target site, thus creating modest efficacy and a high number of adverse effects. Nanoparticles offer promising possibilities when it comes to overcoming the barriers mentioned, showing a better biodistribution and enhanced accumulation. In order to amplify their potential, researchers have used a vast amount of targeting molecules. Tetraspanins are among the molecules that have shown great potential in this area.

## Figures and Tables

**Table 2:** Summary of tumor progression-promoting tetraspanins.

Tetraspanin	Overview
TSPAN8	- Tumor promoter in gastrointestinal cancers (colorectal, liver, pancreatic cancer); - Mechanism of action: progression of cancer cells; - High level expression: advanced stages and poor prognosis; - Lymph node metastases are also found to have high levels of TSPAN8; - Intrahepatic metastasis express TSPAN8 in hepatocellular cancer; - Development of disseminated intravascular coagulation and hemorrhage at the tumor site in pancreatic cancer.
CD151	- Well documented for implication in malignancies; also involved in nephrotic syndrome, primary glomerular disease, and even the process of wound healing; - Mechanism of action: cell invasion and proliferation; - High levels expression: poor prognosis and high recurrence rates; - Its inhibition could potentially be useful for the development of oncologic treatments

**Table 3:** Summary of tumor progression-suppressing tetraspanins.

Tetraspanin	Overview
CD9	- Low expression in metastatic tumors; - Mechanism of action: its knockdown can be correlated with promoting tumoral invasion and progression; - Association of CD9 with various coupling proteins is considered as the mechanism for variate or even opposite effects; - Expression rate: correlation with patients’ survival rates, disease-free interval, and recurrence rates; - Might also be involved in tumor–endothelial cell interaction and therefore contribute to tumor lymphangiogenesis; - Involved in tumoral proliferation and metastasis by suppressing these processes. More research is needed in order to fully understand its role.
CD63	- Mechanism of action: downregulation of CD63 stimulates progression and metastasis; - Expressed in melanomas (early stage); - Repressed levels: lung and ovarian cancer are progressing (advanced stages).
CD82	- Indirectly proportional to the patient’s prognosis in gastric, colorectal, lung, breast bladder, prostate, and endometrial cancer and others; - Mechanism of action: altering the function of different types of molecules, such as integrins, growth factor receptors, c-MET, and uPAR; - High level of expression: increased internalization of the growth factor receptor.
